# Satellite double-stranded RNA induces mesenchymal transition in pancreatic cancer by regulating alternative splicing

**DOI:** 10.1016/j.jbc.2024.105742

**Published:** 2024-02-10

**Authors:** Takuma Iwata, Takahiro Kishikawa, Takahiro Seimiya, Genso Notoya, Tatsunori Suzuki, Chikako Shibata, Yu Miyakawa, Nariaki Odawara, Kazuyoshi Funato, Eri Tanaka, Mari Yamagami, Kazuma Sekiba, Motoyuki Otsuka, Kazuhiko Koike, Mitsuhiro Fujishiro

**Affiliations:** Department of Gastroenterology, Graduate School of Medicine, The University of Tokyo, Tokyo, Japan

**Keywords:** satellite RNA, double-stranded RNA, pancreatic cancer, STRBP, epithelial–mesenchymal transition, alternative splicing

## Abstract

Human satellite II (HSATII), composed of tandem repeats in pericentromeric regions, is aberrantly transcribed in epithelial cancers, particularly pancreatic cancer. Dysregulation of repetitive elements in cancer tissues can facilitate incidental dsRNA formation; however, it remains controversial whether dsRNAs play tumor-promoting or tumor-suppressing roles during cancer progression. Therefore, we focused on the double-stranded formation of HSATII RNA and explored its molecular function. The overexpression of double-stranded HSATII (dsHSATII) RNA promoted mesenchymal-like morphological changes and enhanced the invasiveness of pancreatic cancer cells. We identified an RNA-binding protein, spermatid perinuclear RNA-binding protein (STRBP), which preferentially binds to dsHSATII RNA rather than single-stranded HSATII RNA. The mesenchymal transition of dsHSATII-expressing cells was rescued by STRBP overexpression. Mechanistically, STRBP is involved in the alternative splicing of genes associated with epithelial–mesenchymal transition (EMT). We also confirmed that isoform switching of CLSTN1, driven by dsHSATII overexpression or *STRBP* depletion, induced EMT-like morphological changes. These findings reveal a novel tumor-promoting function of dsHSATII RNA, inducing EMT-like changes and cell invasiveness, thus enhancing our understanding of the biological significance of aberrant expression of satellite arrays in malignant tumors.

Repetitive elements comprise more than half of the human genome and are classified into two types based on their repeating structure: interspersed repetitive DNAs and tandem repeats. While the interspersed repeats are primarily composed of retrotransposons and dispersed throughout the genome, satellite repeats are preferentially located in centromeric and subtelomeric heterochromatin and clustered in long megabase-size arrays (1). Recently, improvements in computational approaches with high-throughput ultralong sequencing by the telomere-to-telomere (T2T) consortium have generated a complete human reference genome map and a precise transcriptional and epigenetic landscape of centromeric and pericentromeric regions (2, 3, 4). The transcription of pericentromeric satellite arrays is epigenetically silenced under physiological conditions because of the recruitment of chromatin modifiers to maintain heterochromatic status; however, the dysregulation of such satellite array expression has been reported in several malignant diseases (1). Human satellite II (HSATII), a dominant human satellite family located in pericentromeric regions, is highly expressed in pancreatic cancer, but not in normal tissues (5). High HSATII RNA levels were detected in the sera of patients with pancreatic cancer compared to those without cancer (6, 7). HSATII expression is also correlated with poor survival rates in ovarian and colorectal cancers (8, 9). Although HSATII RNAs may not be translated into protein, their oncogenic roles, such as inducing cell division deficiency and chromosomal instability (10), maintaining tumor-assisting immune microenvironment (11), or enhancing senescence-associated secretory phenotype in a noncell autonomous manner (12), have been reported.

Transcripts derived from repetitive elements are good resources for endogenous dsRNA formation since repetitive elements are often bidirectionally transcribed *in cis* or *in trans* throughout the genome (13). Centromeric satellite repeats can also be transcribed bidirectionally (14, 15), and it is reported that HSATII RNA is bidirectionally transcribed to form dsRNA in skeletal muscle cells overexpressing double homeobox 4 (DUX4) (16). The increase in endogenous dsRNA in cancer cells is considered tumor-suppressive since dsRNAs are recognized by pattern recognition receptors and trigger an innate immune system-mediated interferon response that mimics antiviral responses (17). Epigenetic therapy triggers the transcription of endogenous repetitive elements and enhances tumor immunity through the activation of antiviral response (18, 19). In contrast, the tumor-promoting roles of dsRNA have recently been reported; tumor-cell migration toward blood vessels is accelerated through intercellular communication between tumor-derived dsRNA and endothelial cells (20), and intrinsic activation of the interferon signature via the dsRNA sensor develops a protumorigenic microenvironment and contributes to poor patient outcomes in pancreatic cancer (21). Collectively, the pathological significance of dsRNAs in cancer progression remains unclear.

In this study, we explored the biological significance of the bidirectional transcripts of HSATII repeats comprising dsRNA in pancreatic cancer progression. Our findings suggest that overexpression of satellite dsRNA induces a mesenchymal-like phenotype in pancreatic cancer cells and activates cell invasiveness. We further identified an RNA binding protein (RBP) that binds to double-stranded HSATII (dsHSATII) RNA and investigated its molecular mechanisms regulating the epithelial–mesenchymal transition (EMT).

## Results

### Bidirectional HSATII RNA and dsRNA were highly expressed in pancreatic cancer

**Figure 1:**
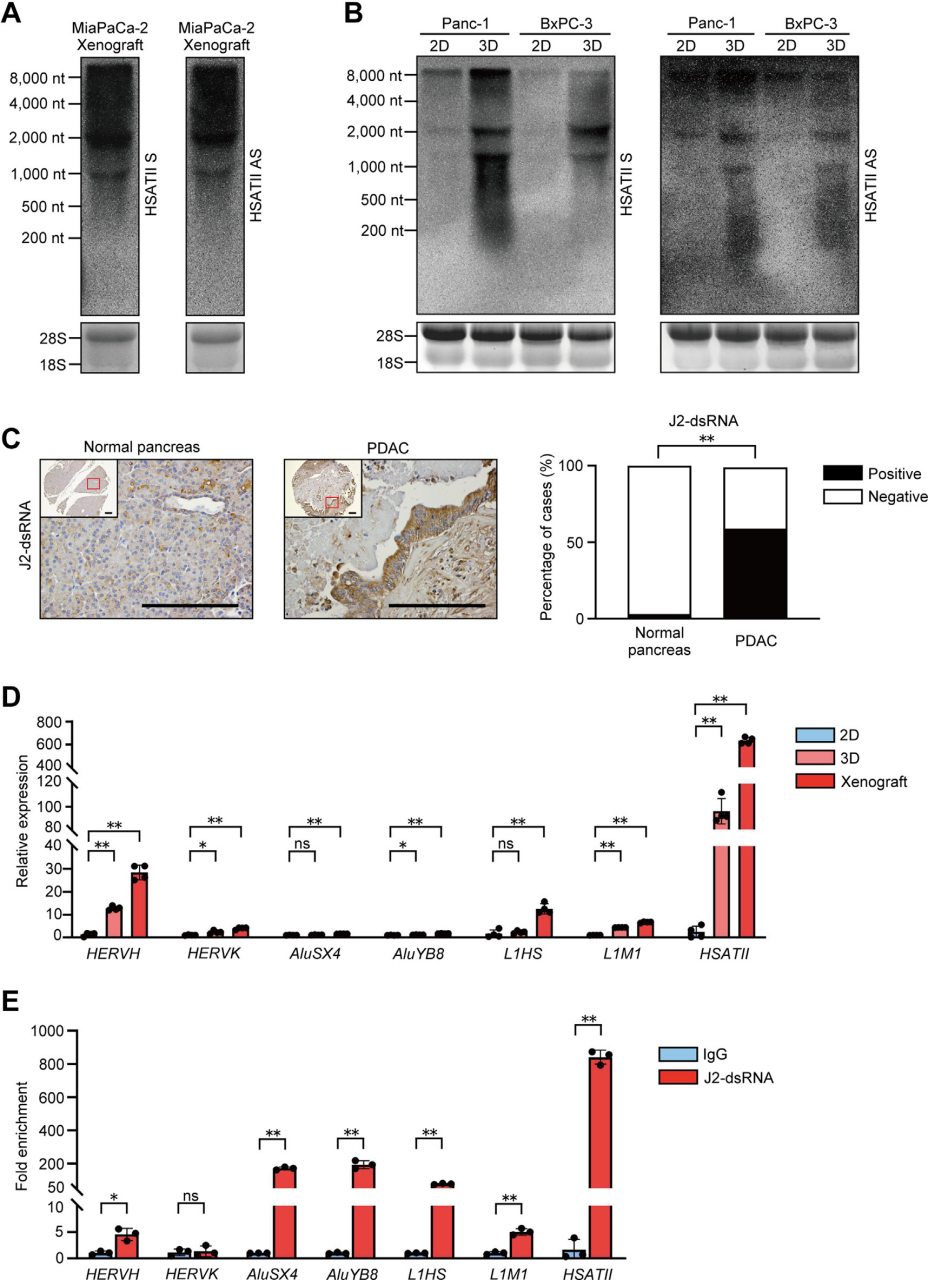
**Bidirectional HSATII RNA and dsRNA were highly expressed in pancreatic cancer.***A*, Northern blotting images of both sense (S) and antisense (AS) HSATII transcripts in xenografts of MiaPaCa-2 cells. *B*, Northern blotting of HSATII RNA in Panc-1 and BxPC-3 cells grown as adherent cultures (2D) or tumorspheres (3D). 18S and 28S rRNA were visualized using ethidium bromide staining as a loading control. *C*, (*left*) representative images of immunohistochemistry stained with anti-J2-dsRNA antibody. The scale bars represent 400 μm. (*Right*) comparison of the positivity rate of dsRNA between normal pancreas and pancreatic ductal adenocarcinoma (PDAC). Normal pancreas: n = 100, PDAC: n = 99. (Fisher’s exact test). *D*, RT-qPCR assay for the expression levels of various repetitive sequences in Panc-1 cells cultured under 2D, 3D, and xenograft conditions. Data are shown as mean ± S.D. of quadruplicate experiments. *E*, RNA immunoprecipitation assay on Panc-1 xenografts using anti-J2-dsRNA antibody. Immunoprecipitated RNA was analyzed by RT-qPCR. Data are shown as mean ± S.D. of triplicate experiments. ∗∗*p* < 0.01, ∗*p* < 0.05, ns: not significant (Student’s *t* test). HSATII, human satellite II; RT-qPCR, reverse transcription and quantitative PCR.

HSATII repeats are expressed aberrantly in pancreatic cancer compared with normal pancreas (5). To investigate whether transcription occurs bidirectionally in pancreatic cancer tissues, we examined endogenous HSATII expression in both sense (S) and antisense (AS) strands through Northern blotting using complementary probes specific for HSATII RNA. Since HSATII expression is induced under 3D cultured conditions such as xenografts and tumor spheres, and silenced under adherent culture conditions (2D) (5, 9), we evaluated human pancreatic cancer cell lines cultured in 3D settings. Bidirectional transcription of HSATII repeats in MiaPaCa-2 xenografts and 3D-cultured Panc-1 and BxPC-3 cells was detected as smear bands, indicating that HSATII RNA is composed of various heterogeneous transcripts derived from multiple genomic loci (Fig. 1, *A* and *B*). As previously reported, HSATII expression was nearly undetectable in 2D-cultured cells (6, 9). Hence, we hypothesized that both strands of HSATII RNA hybridize each other and form RNA duplexes in pancreatic cancer tissues. Since direct detection of the double-stranded formation of HSATII RNA using RNA *in situ* hybridization posed technical challenges, we opted to investigate the expression levels of dsRNA in human pancreatic cancer tissues and compared them with those in normal pancreatic tissues. Immunohistochemistry analysis using anti-J2-dsRNA antibody revealed significantly higher levels of dsRNA expression in cancerous pancreatic ductal epithelial cells than in normal pancreatic ductal cells (Fig. 1*C*), consistent with previous reports (21, 22). Of the 99 samples, 59 (59.6%) were positive for pancreatic cancer tissue, whereas three of 100 samples (3%) were positive for normal pancreatic tissue. Subsequently, to investigate the proportion of dsHSATII RNA within dsRNAs expressed in tumors, the expression levels of variable repeat families in Panc-1 cells cultured in 2D, 3D, and xenograft settings were evaluated. Reverse transcription and quantitative-PCR (RT-qPCR) analyses showed that HSATII RNA expression was most significantly upregulated in 3D-cultured cells and xenografts compared to the 2D setting. The upregulation of other repetitive elements, such as endogenous retroviruses (ERVs), short interspersed nuclear elements (SINEs), and long interspersed nuclear elements (LINEs), was not as remarkable as that of HSATII (Fig. 1*D*). The data suggest that tumor-specific dysregulation of HSATII RNA derived from heterochromatin regions could lead to the abnormal accumulation of dsRNA. Furthermore, RNA immunoprecipitation using anti-J2-dsRNA antibody in Panc-1 xenografts demonstrated that HSATII RNA exhibited the highest enrichment among these repetitive arrays (Fig. 1*E*), suggesting that HSATII, composed of highly tandem repeats of relatively short array, has a propensity to form dsRNA compared to the interspersed repeat elements. These results indicate that dysregulated bidirectional HSATII transcription can provide a resource that can facilitate dsRNA formation in pancreatic tumors.

### HSATII RNA-derived dsRNA promoted a mesenchymal phenotype

**Figure 2:**
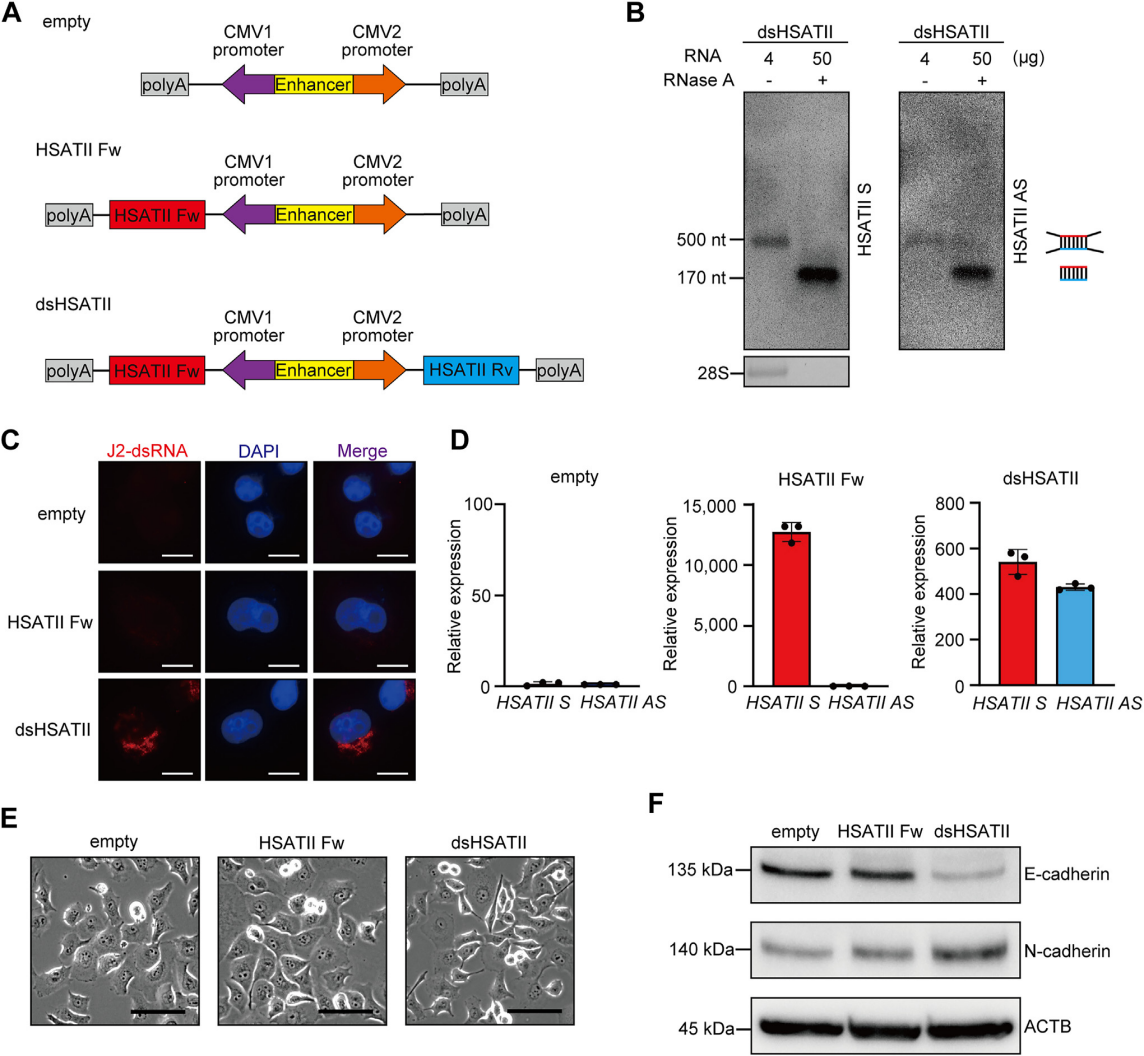
**Phenotypical changes in Panc-1 cells overexpressing dsHSATII RNA.***A*, schematic diagram of vectors for bidirectional transcription of HSATII. Both HSATII Fw (forward strand) and HSATII Rv (reverse strand) sequences were transcribed under two divergent CMV promoters. dsRNA was formed by 170 nt of complementary sequences in HSATII Fw and Rv transcripts. *B*, total RNA was extracted from Panc-1 cells transiently transfected with bidirectional HSATII RNA, and treated with RNase A to digest flanking noncomplementary parts. Northern blotting shows intracellular dsRNA formation at 170 nt, protected from RNase A digestion. The 28S rRNA band was used as a loading control. *C*, immunofluorescence cytochemistry using anti-J2-dsRNA antibody in Panc-1 cells transiently transfected with the pBI-dsHSATII vector. The scale bars represent 10 μm. *D*, relative expression levels of HSATII Fw and Rv RNA were quantified using RT-qPCR in Panc-1 cells stably expressing HSATII Fw and dsHSATII RNA. Data are shown as mean ± S.D. of triplicate experiments. *E*, phase-contrast images showing fibroblast-like morphological changes in Panc-1 dsHSATII cells. The scale bars represent 50 μm. *F*, Western blotting of epithelial and mesenchymal markers in Panc-1 cells. ACTB was used as a loading control. ACTB, actin beta; CMV, cytomegalovirus; dsHSATII, double-stranded HSATII; HSATII, human satellite II; HSATII Fw, HSATII forward strand; HSATII Rv, HSATII reverse strand; RT-qPCR, reverse transcription and quantitative PCR.

To elucidate the biological effects of dsRNA formation derived from HSATII repeats on cancer progression, we established an *in vitro* assay model with pancreatic cancer cells stably expressing dsHSATII RNA. The HSATII forward strand (HSATII Fw) was derived from a consensus HSATII array of 170 bp, whereas the HSATII reverse strand (HSATII Rv) was derived from its complementary sequence. Each HSATII Fw and HSATII Rv sequence was inserted under two divergent cytomegalovirus (CMV) promoters in the pBI-CMV vector for bidirectional transcription (pBI-dsHSATII, Fig. 2*A*). As an ssRNA control, pBI-HSATII Fw vector was constructed to include only the HSATII Fw sequence. They were transfected into Panc-1 cells in which endogenous HSATII RNA expression is negligible under 2D conditions (Fig. 1*B*). To confirm the intracellular dsRNA formation of HSATII, we first extracted total RNA from Panc-1 cells transiently transfected with the pBI-dsHSATII vector. Subsequently, the RNA samples were treated with RNase A, with the aim of preserving a 170-base pair complete dsRNA, while digesting the flanking noncomplementary regions. Northern blotting showed the presence of 170 base bands both in HSATII S and HSATII AS after RNase A treatment, suggesting that the complementary part of each strand forms dsRNA in the cells (Fig. 2*B*). We validated the intracellular formation of dsHSATII RNA by immunofluorescence cytochemistry using anti-J2-dsRNA antibody (Fig. 2*C*). Subsequently, Panc-1 cells that stably express HSATII Fw or dsHSATII RNA (Panc-1 HSATII Fw and Panc-1 dsHSATII, respectively) were established. RT-qPCR confirmed higher HSATII expression levels in Panc-1 HSATII Fw and Panc-1 dsHSATII cells than in Panc-1 empty cells (Fig. 2*D*). Remarkably, Panc-1 dsHSATII cells, but not Panc-1 HSATII Fw cells, underwent morphological changes, transitioning from cobblestone-like epithelial cells to spindle-shaped mesenchymal cells (Fig. 2*E*). Additionally, a decrease in the epithelial marker E-cadherin and an increase in the mesenchymal marker N-cadherin were observed in Panc-1 dsHSATII cells by Western blotting (Fig. 2*F*). To validate the morphological cell alterations above induced by dsHSATII RNA expression, BxPC-3 and MiaPaCa-2 cells stably expressing dsHSATII RNA were established (Fig. S1, *A* and *D*). Consistent with Panc-1 cells, both cell lines exhibited spindle-like morphological changes and shifts toward mesenchymal expression profiles with dsHSATII RNA expression in contrast to single-stranded HSATII (ssHSATII) RNA (Fig. S1, *B*, *C*, *E*, and *F*).

**Figure 3:**
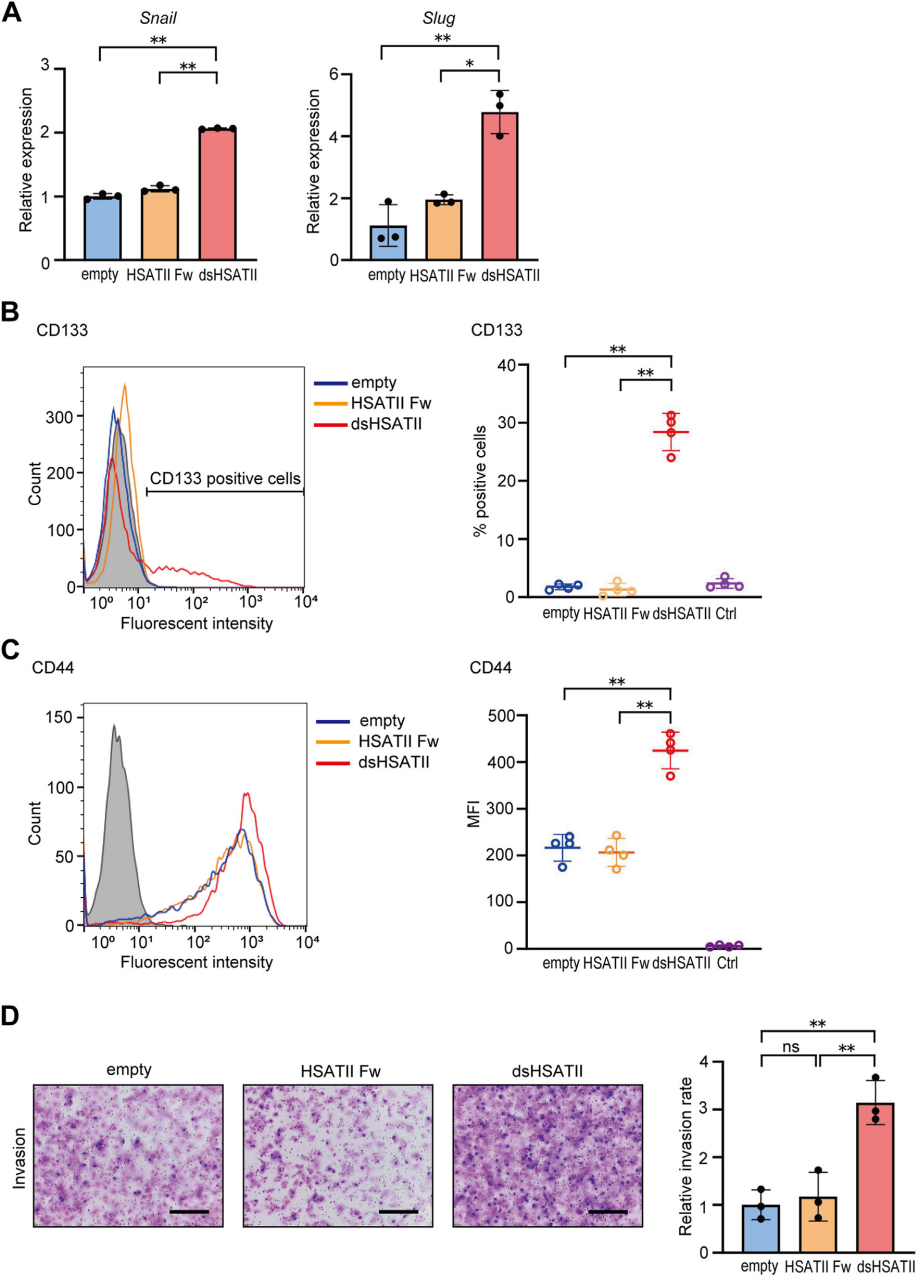
**Overexpression of dsHSATII induced EMT-like changes in Panc-1 cells.***A*, the expression profiles of transcriptional factors associated with the regulation of EMT. Total RNA from Panc-1 empty, HSATII Fw, and dsHSATII cells were analyzed by RT-qPCR. Data are shown as mean ± S.D. of triplicate experiments. *B*, the expression profiles of CD133 in Panc-1 cells expressing dsHSATII RNA. The percentage of cells positively stained with anti-CD133 antibody was quantified and plotted in the right panel. Data are shown as mean ± S.D. from quadruplicate assays. *C*, the expression profiles of CD44 were assessed by flow cytometry. Mean fluorescence intensities (MFI) were plotted in the right panel. Data are shown as mean ± S.D. from quadruplicate assays. *D*, (*left*) transwell invasion assay in Panc-1 cells expressing HSATII Fw or dsHSATII RNA. Cells (5 × 10^5^) were incubated for 48 h. The scale bars represent 300 μm. (*Right*) the relative rate of invading cells was quantified. Data are shown as mean ± S.D. from triplicate assays. ∗*p* < 0.05, ∗∗*p* < 0.01, ns: not significant (one-way ANOVA followed by Tukey’s multiple comparisons test). dsHSATII, double-stranded HSATII; HSATII, human satellite II; HSATII Fw, HSATII forward strand; EMT, epithelial–mesenchymal transition; RT-qPCR, reverse transcription and quantitative PCR.

To further investigate the phenotypical characteristics of dsHSATII RNA expressing cells, the expression levels of transcriptional factors regulating the EMT process were assessed. *Snail* and *Slug* were also upregulated in Panc-1 dsHSATII cells rather than in Panc-1 empty and HSATII Fw cells (Fig. 3*A*). Subsequently, the subpopulation of cells undergoing mesenchymal transition was investigated by examining the expression of CD133, recognized as a potential marker for EMT and cancer stemness in various cancers, including pancreatic cancer (23, 24). Flow cytometry analyses revealed that 28.4% of Panc-1 dsHSATII cells were positively stained with anti-CD133 antibody, whereas Panc-1 empty and HSATII Fw cells were almost negative (Fig. 3*B*). Additionally, the expression levels of CD44, another cancer stem cell marker upregulated in pancreatic cancer cells undergoing EMT (25), were elevated in Panc-1 dsHSATII cells compared to in Panc-1 empty and HSATII Fw cells (Fig. 3*C*). Finally, Transwell Matrigel invasion assays were performed to evaluate the cell invasiveness of each cell line. Panc-1 dsHSATII cells showed enhanced invasive capacities compared to the control, whereas single-stranded expression did not change invasiveness (Fig. 3*D*). Collectively, these findings indicate that the expression of dsHSATII RNA induces characteristic changes associated with EMT in pancreatic cancer cells.

To evaluate whether the phenotypic changes induced by dsHSATII RNA are sequence specific, bidirectional expression vectors containing other types of repetitive arrays were generated; HERVH (ERV), AluSX (SINE), and L1M1 (LINE), followed by the establishment of Panc-1 cells stably expressing the dsRNA derived from these repetitive elements (Fig. S2*A*). A shift in cell morphology and altered expression patterns of EMT marker proteins were observed only in cells expressing double-stranded L1M1, while they were not detected in the other two repeats (Fig. S2, *B* and *C*). Notably, the extents of the changes were weaker compared to that in the Panc-1 dsHSATII cells. The findings suggest that the induction of EMT-like state by the formation of dsRNA is not entirely specific to the HSATII sequence, but there is some inclination within repeat families.

### dsHSATII RNA bound to spermatid perinuclear RNA-binding protein (STRBP)

**Figure 4:**
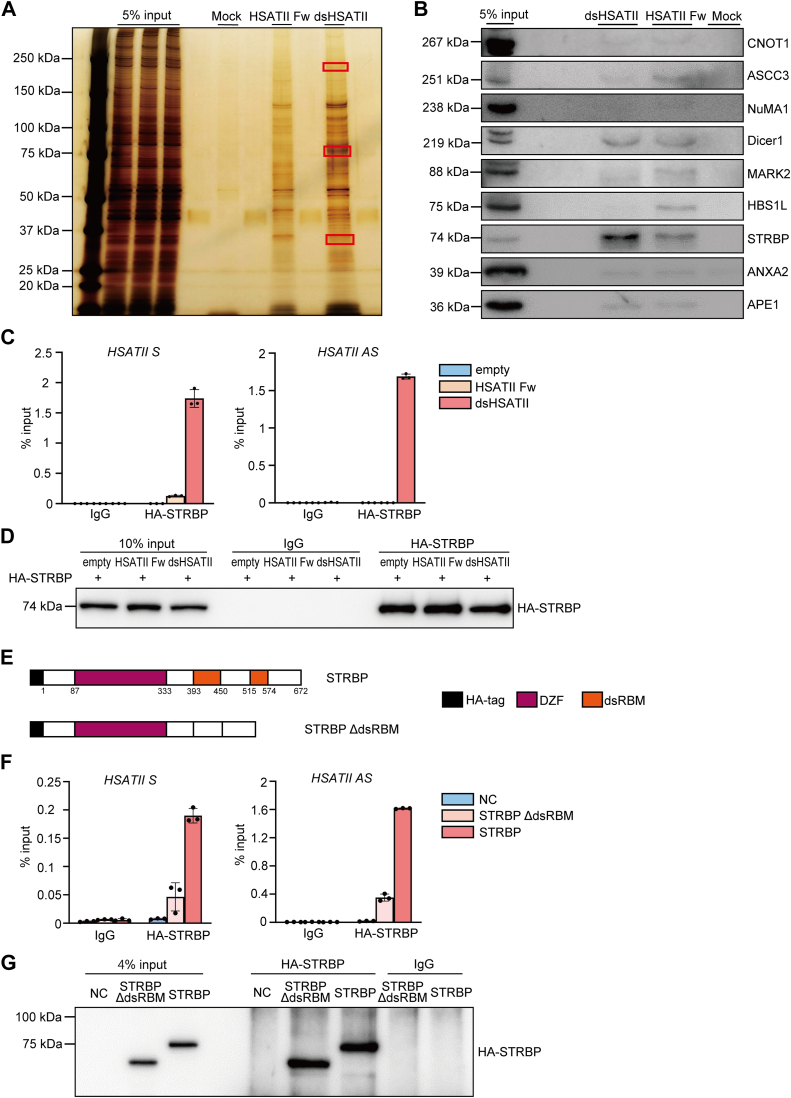
**HSATII dsRNA bound to STRBP.***A*, total lysates from Panc-1 cells were incubated with a biotinylated dsHSATII RNA probe, and pulled down with streptavidin beads. HSATII Fw RNA was used as a control. The resulting precipitates were separated by gel electrophoresis and visualized through silver staining. Specific bands identified in dsHSATII lane, marked by the three *red boxes*, were subsequently analyzed using LC-MS/MS. *B*, Western blotting analyses of precipitates pulled down by dsHSATII RNA probe to confirm the binding of candidate RNA-binding proteins based on LC-MS/MS results. *C*, RNA immunoprecipitation assay was performed on HEK293T cells using anti-HA-tag antibody. Cells were transiently transfected with vectors expressing HA-STRBP and either pBI-HSATII Fw or pBI-dsHSATII RNA. The enrichment of each strand of HSATII RNA was assessed by RT-qPCR. Data are shown as mean ± S.D. from triplicate assays. *D*, Western blotting image of the immunoprecipitated HA-STRBP. *E*, schematic images of STRBP and its deletion mutant. DZF: domain associated with zinc fingers. dsRBM: double-stranded RNA binding motif. *F*, the lysates of HEK293T cells cotransfected with pBI-dsHSATII and either HA-STRBP or HA-STRBP ΔdsRBM vector were subjected to RNA immunoprecipitation using anti-HA-tag antibody. Both strands of HSATII RNA in the immunoprecipitates were analyzed with RT-qPCR and plotted as a percentage of the input. Data are shown as mean ± S.D. from triplicate assays. *G*, Western blotting image of the immunoprecipitated HA-STRBP. dsHSATII, double-stranded HSATII; dsRBM, double-stranded RNA binding motifs; HA, hemagglutinin; HSATII, human satellite II; HSATII Fw, HSATII forward strand; LC-MS/MS, liquid chromatography–tandem mass spectrometry; RT-qPCR, reverse transcription and quantitative PCR; STRBP, spermatid perinuclear RNA-binding protein.

Long noncoding RNAs often play oncogenic roles in collaboration with specific binding proteins (26). Several studies focusing on the interaction between repetitive satellite RNAs and their binding proteins have been reported (27, 28, 29). ssHSATII RNA is involved in colorectal tumorigenesis by interacting with methyl-CpG binding protein 2 (MECP2) (10) and CCCTC-binding factor (CTCF) (12). Therefore, we sought to identify RBPs specifically binding to dsHSATII RNA to elucidate the molecular mechanisms underlying EMT-like morphological changes. To conduct an RNA pull-down assay using dsHSATII RNA probe as bait, we annealed each strand of HSATII RNA oligonucleotides and confirmed the formation of dsHSATII oligonucleotides by digesting ssRNA with S1 nuclease. Mops-formaldehyde gel electrophoresis showed that 170 bp of complementary HSATII sequence was preserved, whereas flanking noncomplementary parts were digested by S1 nuclease (Fig. S3*A*). Subsequently, RNA pull-down assay was performed using total lysates from Panc-1 cells and biotinylated 170-nucleotides (nt) dsHSATII RNA. We used 264 nt HSATII Fw as a control for ssRNA. Three bands specifically detected in dsHSATII compared to HSATII Fw were collected and subjected to liquid chromatography–tandem mass spectrometry (LC-MS/MS) (Fig. 4*A*). From the proteins identified through LC-MS/MS analysis, nine candidate RBPs annotated with terms as “RNA-binding” or “double-stranded RNA binding” in the Gene Ontology annotation were selected (Table S1). Subsequently, Western blotting assay was performed to confirm the binding of the candidate proteins. We identified spermatid perinuclear RNA-binding protein (STRBP) that showed a stronger binding to dsHSATII compared to ssHSATII oligonucleotides, whereas the other candidates showed nonenrichment (CNOT1, NuMA1, and ANXA2) or nonspecific binding (ASCC3, Dicer1, MARK2, HBS1L, and APE1) to both dsHSATII and HSATII Fw (Fig. 4*B*). Conversely, we conducted an RNA immunoprecipitation assay using HEK293T cells overexpressing hemagglutinin (HA)-tagged STRBP (HA-STRBP) and either dsHSATII or HSATII Fw RNA. The immunoprecipitates with anti-HA-tag antibody exhibited the enrichment of both strands of HSATII RNA in dsHSATII-expressing cells while ssHSATII RNA was not enriched (Fig. 4, *C* and *D*). The binding was further confirmed in Panc-1 cells expressing dsHSATII RNA and HA-tagged STRBP (Fig. S3, *B* and *C*). Afterward, the binding efficacies of other types of repetitive sequences to STRBP were evaluated. RT-qPCR results showed that all repeat sequences were enriched in the immunoprecipitates with anti-HA-tag antibody, but HSATII exhibited the highest enrichment among them (Fig. S3*D*). Overall, the data indicate that STRBP exhibits a higher affinity for dsHSATII RNA rather than ssHSATII RNA or other repetitive RNAs.

Since STRBP possesses two double-stranded RNA binding motifs (dsRBMs) (30), expression vectors for expressing HA-STRBP in full length and its deletion mutant lacking the dsRBMs (HA-STRBP ΔdsRBM) were generated (Fig. 4*E*). Each vector was transiently cotransfected into HEK293T cells with pBI-dsHSATII vector. RNA immunoprecipitation assay using anti-HA-tag antibody showed that the binding efficacy of dsHSATII RNA was reduced in STRBP ΔdsRBM expressing cells (Fig. 4, *F* and *G*). The findings suggest that dsHSATII RNA is bound to STRBP through the dsRBMs.

### Depletion of *STRBP* induced EMT-like morphological changes

**Figure 5:**
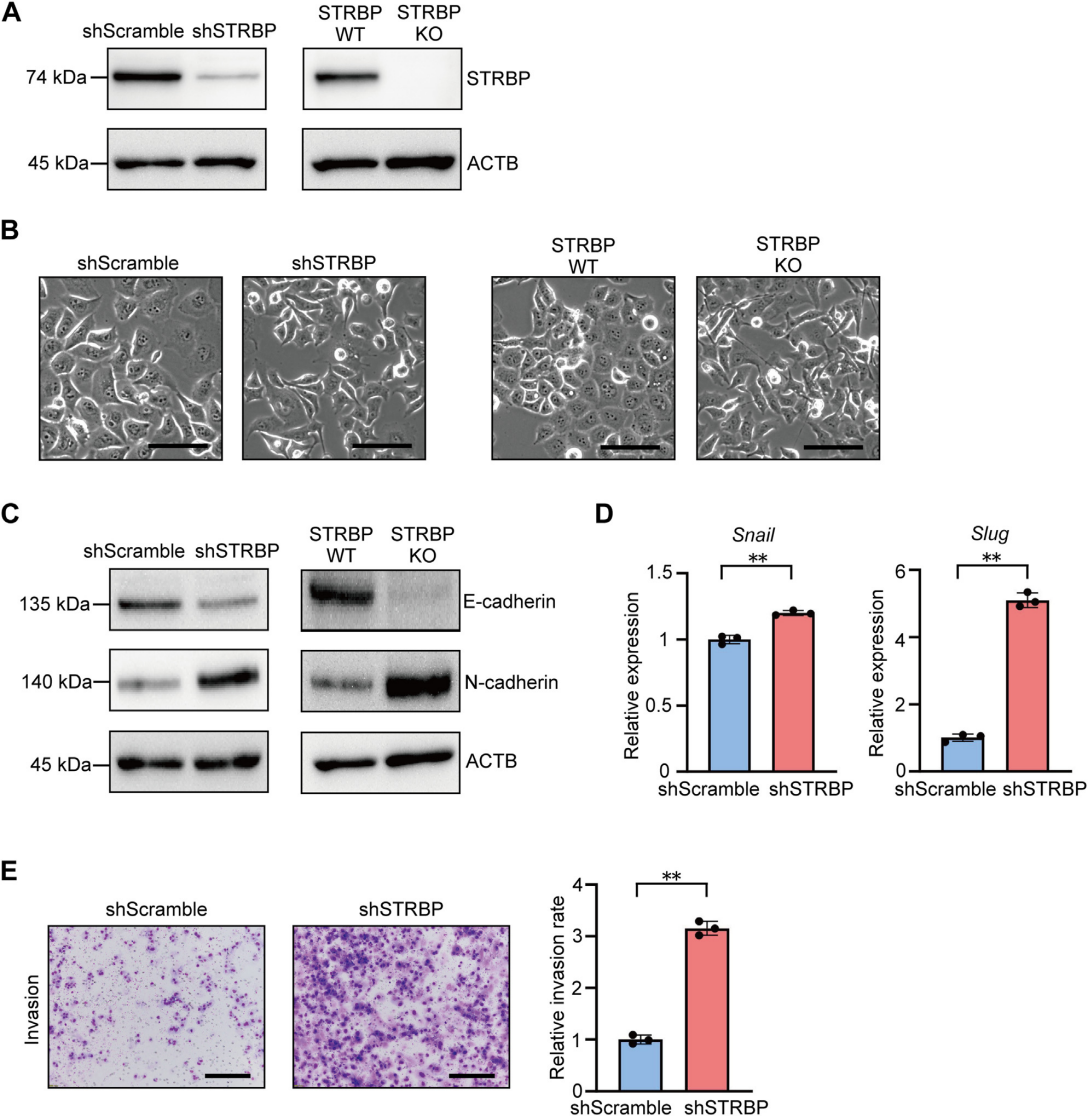
**Depletion of *STRBP* induced EMT-like morphological change.***A*, *STRBP* knockdown (KD) and KO in Panc-1 cells were confirmed by Western blotting compared to controls. *B*, morphological changes in Panc-1 STRBP KD and KO cells are shown as phase-contrast images. The scale bars represent 50 μm. *C*, Western blotting showing EMT markers in Panc-1 STRBP KD or KO cells. *D*, relative expression levels of *Snail* and *Slug* in Panc-1 STRBP KD cells. Data are shown as mean ± S.D. from triplicate assays. *E*, (*left*) transwell invasion assay was performed in Panc-1 shScramble or Panc-1 STRBP KD cells. The scale bars represents 300 μm. (*Right*) the relative rate of invading cells was quantified. Data are shown as mean ± S.D. from triplicate assays. ∗∗*p* < 0.01 (Student’s *t* test). EMT, epithelial–mesenchymal transition; KD, knockdown; STRBP, spermatid perinuclear RNA-binding protein.

STRBP is involved in spermatogenesis and sperm mobility (31, 32, 33); however, few studies have explored its roles in malignant diseases (34). We hypothesized that the interaction between STRBP and dsHSATII RNA would cause imbalance in the cellular epithelial and mesenchymal status. To evaluate whether the EMT-like changes are induced by *STRBP* depletion in pancreatic cancer cells, we established *STRBP* knockdown (KD) cells stably expressing shRNA for *STRBP* and *STRBP* KO cells in which the genomic locus of *STRBP* was deleted using the CRISPR-Cas9 system. Both *STRBP* KD and KO induced morphological changes and increased the expression of mesenchymal marker in Panc-1 cells (Figs. 5, *A*–*D*, and S4, *A* and *B*). We further found that Panc-1 STRBP KD cells acquired invasive capacity (Fig. 5*E*). Notably, such characteristics were consistent with those observed in Panc-1 dsHSATII cells (Fig. 2).

**Figure 6:**
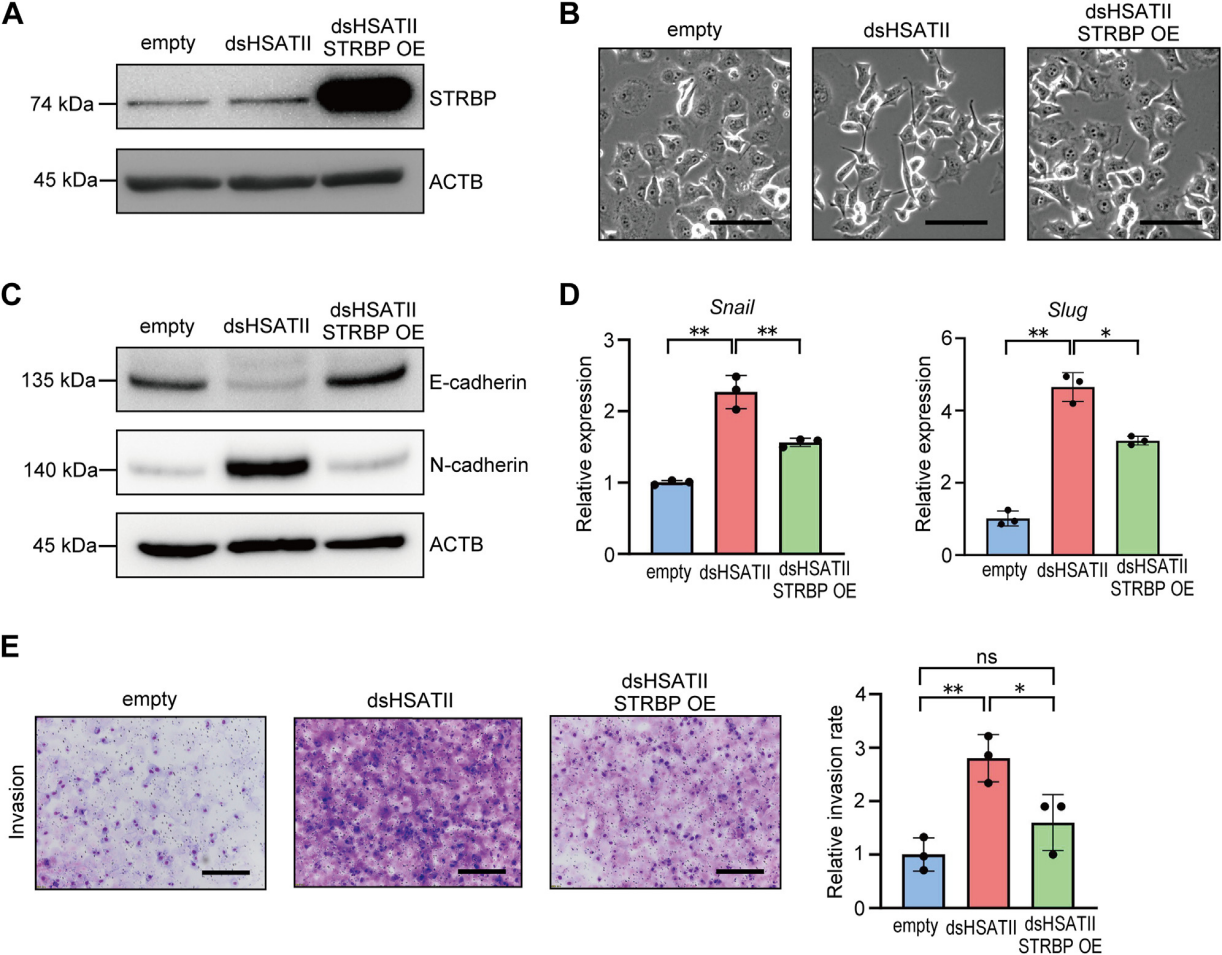
**STRBP overexpression reverted the mesenchymal phenotype in Panc-1 dsHSATII cells.***A*, confirmation of STRBP overexpression (OE) in Panc-1 dsHSATII cells. *B*, phase contrast images indicate that morphological changes were induced by STRBP OE in Panc-1 dsHSATII cells. The scale bars represent 50 μm. *C*, Western blotting of EMT markers in Panc-1 cells stably expressing dsHSATII and STRBP. *D*, relative expression levels of *Snail* and *Slug* in Panc-1 dsHSATII-STRBP OE cells. Data are shown as mean ± S.D. from triplicate assays. *E*, (*left*) transwell invasion assay in Panc-1 cells expressing dsHSATII and STRBP. The scale bars represent 300 μm. (*Right*) the relative invasion rate is shown as mean ± S.D. from triplicate assays. ∗*p* < 0.05, ∗∗*p* < 0.01, ns: not significant (one-way ANOVA followed by Tukey’s multiple comparisons test). dsHSATII, double-stranded HSATII; EMT, epithelial–mesenchymal transition; HSATII, human satellite II; STRBP OE, STRBP overexpression; STRBP, spermatid perinuclear RNA-binding protein.

Based on the observations above, we next investigated whether Panc-1 dsHSATII cells underwent mesenchymal to epithelial reversal by overexpressing STRBP (Fig. 6*A*). The spindle-like morphological changes in Panc-1 dsHSATII cells were partially reversed by STRBP overexpression (Fig. 6*B*). Mesenchymal expression profile and invasive potential were also suppressed in Panc-1 dsHSATII-STRBP overexpression cells (Figs. 6, *C*–*E* and S4, *C* and *D*). STRBP expression levels were not decreased in Panc-1 dsHSATII cells, indicating that the induction of EMT-like phenotypes by dsHSATII expression is partly dependent on the dysfunction of STRBP bound to dsHSATII RNA, rather than a decrease in STRBP expression (Fig. 6*A*).

### STRBP regulated EMT-associated alternative splicing events

**Figure 7:**
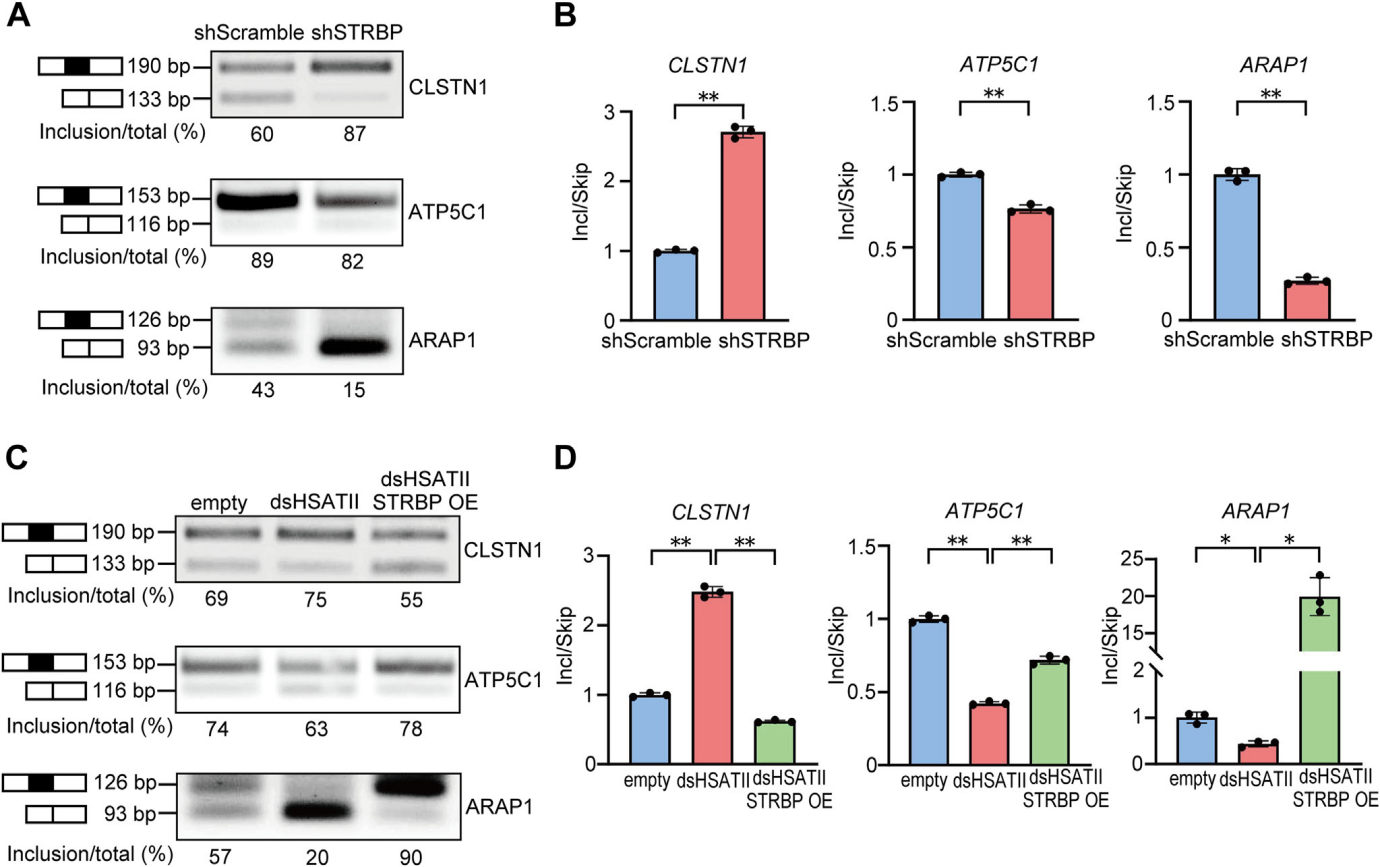
**STRBP regulated EMT-associated alternative splicing events.***A*, semi-qPCR was performed to quantify alternative splicing events for EMT-associated genes. The upper and lower bands for each gene indicate included and skipped splicing products, respectively. Inclusion/total was the proportion of exon inclusion out of the total splicing event. *B*, RT-qPCR assays were performed using isoform-specific primers and the ratio of exon inclusion to exon skipping was plotted as mean ± S.D. from triplicate assays (Student’s *t* test). Mesenchymal-favoring isoforms increased in *STRBP*-depleted Panc-1 cells. *C*, semi-qPCR analyses for each splicing variant in Panc-1 dsHSATII-STRBP OE cells. *D*, RT-qPCR analyses using isoform specific primers. Data are shown as mean ± S.D. from triplicate assays. ∗*p* < 0.05, ∗∗*p* < 0.01 (Bonferroni’s test). dsHSATII, double-stranded HSATII; EMT, epithelial–mesenchymal transition; HSATII, human satellite II; RT-qPCR, reverse transcription and quantitative PCR; STRBP, spermatid perinuclear RNA-binding protein; STRBP OE, STRBP overexpression.

Several studies have reported that RBPs modify EMT status by directly regulating alternative splicing events (35, 36). Recent computational analyses have predicted alternative splicing-associated RBPs, including STRBP, which are associated with splicing dynamics during EMT (37). To validate the alternation of splicing patterns *in vitro*, we selected three genes in which alternative splicing events were predicted during EMT: the skipping of *CLSTN1* exon 11, the inclusion of *ATP5C1* exon 9, and the inclusion of *ARAP1* exon 25. We investigated the splicing pattern of each gene in *STRBP* KD cells using semiquantitative RT-PCR and RT-qPCR. The ratios of exon inclusion to skipping shifted to mesenchymal-favoring patterns in the cells (Fig. 7, *A* and *B*). Moreover, it reversed to epithelial-favoring splicing patterns by overexpressing STRBP in Panc-1 dsHSATII cells, suggesting that STRBP is involved in regulation of alternative splicing events in EMT-associated genes, which can be impaired by the binding of dsHSATII RNA (Fig. 7, *C* and *D*).

Additionally, we examined the function of STRBP in Panc-1 cells cultured in 3D settings or xenografts, where endogenous dsHSATII RNA was highly expressed (Fig. 1). The alternative splicing patterns shifted toward a mesenchymal status in the cells cultured in 3D and xenograft settings compared to those in 2D culture, whereas the expression levels of STRBP remained unchanged among them (Fig. S5, *A* and *B*). The data suggest that endogenous dsHSATII RNA may influence STRBP function without altering its expression level.

**Figure 8:**
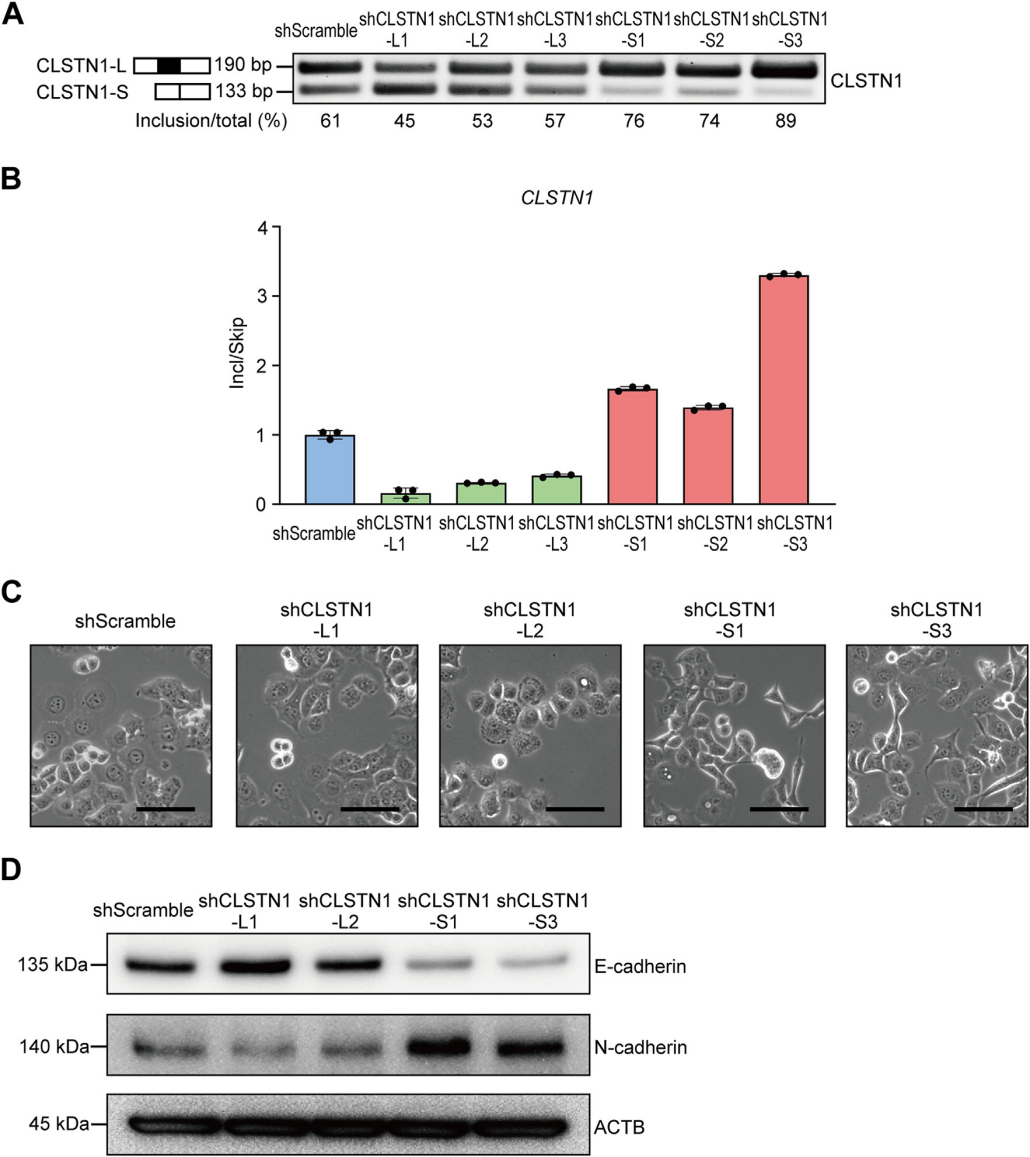
**Isoform specific knockdown of CLSTN1 induced EMT-like phenotypic changes.***A* and *B*, knockdown (KD) of a specific isoform of *CLSTN1* in Panc-1 cells was confirmed by semi-qPCR (*A*) and RT-qPCR (*B*) compared to the shScramble cells. *C*, phase contrast images in Panc-1 CLSTN1-S KD and CLSTN-L KD cells. The scale bars represent 50 μm. *D*, Western blotting images for EMT markers in Panc-1 CLSTN1-S KD and CLSTN-L KD cells. EMT, epithelial–mesenchymal transition; KD, knockdown; RT-qPCR, reverse transcription and quantitative PCR.

Finally, we explored the biological role of isoform switching of EMT-associated genes in the context of mesenchymal transition. We selected *CLSTN1* gene based on previous reports suggesting that an increase in the exon-including isoform of *CLSTN1* (*CLSTN1-L*) is associated with metastasis and poor prognosis in breast and gastric cancers (36, 38). To evaluate the roles of each isoform, we applied isoform-specific shRNAs to silence *CLSTN1-L* or *CLSTN1-S* (exon-skipped short isoform) without reducing the expression of nontargeted isoforms (Fig. 8, *A* and *B*) (36). KD of *CLSTN1-S* in Panc-1 cells resulted in mesenchymal morphological changes and expression profiles. Conversely, *CLSTN1-L* KD cells showed upregulation of E-cadherin and reduction of N-cadherin, although these changes did not result in significant morphological alternations (Fig. 8, *C* and *D*). These results suggest that isoform switching of *CLSTN1* could regulate epithelial and mesenchymal state, which was mediated by STRBP.

## Discussion

In the present study, we showed that HSATII RNA was bidirectionally expressed in pancreatic cancer cells in 3D-cultured conditions, which enabled the formation of dsRNA, and indicated that ectopic overexpression of dsHSATII RNA could promote mesenchymal phenotypes. We identified STRBP that is preferentially bound to the HSATII RNA duplex and observed that its overexpression could rescue the mesenchymal transition of dsHSATII-expressing cells. Furthermore, these phenotypes were related to alternative splicing in EMT-associated genes regulated by the interaction between dsHSATII RNA and STRBP. To the best of our knowledge, this study is the first to report the molecular function of HSATII-derived dsRNA and show its cell-autonomous roles in enhancing cell malignancy.

We did not directly demonstrate the aberrant expression of dsHSATII RNA in pancreatic cancer specimens due to technical challenges in hybridizing tandem repeat dsRNA. Alternatively, we demonstrated tumor-specific dysregulation of HSATII RNA in xenograft tumors, which could lead to the abnormal accumulation of dsRNA. Additionally, HSATII RNA, characterized by a high density of tandem repeats with relatively short arrays, is likely to exhibit a higher tendency to form dsRNA compared to interspersed repeat elements with longer repeat units (16), as shown by RNA immunoprecipitation assay using anti-J2-dsRNA antibody. Collectively, considering tumor-specific dysregulation of HSATII RNA and its propensity to form dsRNA, dsHSATII may have a greater impact on the phenotypical changes in tumor settings.

Our findings indicate that the induction of EMT-like phenotypic changes driven by dsHSATII RNA through the interaction of STRBP may not be strictly sequence specific, at least under ectopic overexpressing conditions. However, a previous report has asserted that the elevation of HSATII RNA, in contrast to other repetitive sequences, such as ERV, SINE, and LINE, induces EMT in cancer cells and is correlated with poor prognosis of epithelial ovarian cancer, which indicates the sequence-specific or tandem structure-specific role of satellite RNA (8). Although the occurrence of bidirectional transcription has not been mentioned, it is plausible that bidirectional transcription and duplex formation of HSATII RNA occur in cancer tissues and drive the EMT signature.

Tumor metastasis is a major cause of cancer death, and the EMT process is closely related to cancer progression to metastasis. In addition to well-known regulators like transcription factors and microRNAs (39), it becomes increasingly evident that the regulation of alternative splicing also contributes to EMT through interaction with specific RBPs (35, 40). Alternative splicing expands mRNA complexity and proteomic diversity by including or excluding exons that encode functional domains (41). For instance, switching CD44 expression from the variant isoform (CD44v) to the standard isoform (CD44s) induced by hnRNPM protein plays a critical role in accelerating EMT in breast cancer by activating Akt signaling (35, 42). STRBP has also been predicted to promote an epithelial state by regulating alternative splicing events of various EMT-associated genes, among which we selected and investigated *CLSTN1*, *ATP5C1*, and *ARAP1* (36, 37). STRBP is expressed broadly in the testis, brain, and other tissues and plays a critical role in human spermatogenesis; loss of *STRBP* causes male infertility and premature death (31, 32, 33). Structurally, STRBP is a dsRBP containing two dsRBMs and one domain associated with zinc fingers (DZF). Indeed, interleukin enhancer binding factor 3, a paralogue of *STRBP*, competes with adenosine deaminase acting on RNA (ADAR) for binding to dsRNAs through its dsRBM structures, thereby impairing ADAR function in A-to-I editing (43). Consistently, we demonstrated that STRBP binds preferentially to dsHSATII RNA rather than ssHSATII RNA through dsRBMs. Although further clarification of the molecular mechanisms is required, STRBP may regulate the splicing machinery in multiple genes by interacting with splicing factors or intronic hairpin dsRNAs.

CLSTN1 is a single-pass type I transmembrane protein belonging to the cadherin superfamily, involved in synapse formation and synaptic plasticity, functioning as a cell adhesion molecule in neuronal cells (44). However, detailed mechanisms substantiating functional changes upon each isoform have not been reported at the moment. In the present study, depletion of the *CLSTN1* short isoform was observed to promote mesenchymal phenotypes in pancreatic cancer. Consistent with our findings, two previous studies have shown that the long isoform of CLSTN1 induces mesenchymal transition and invasiveness (36, 38), suggesting the role of CLSTN1 isoform switching in promoting malignant potential in various cancers.

We have identified a novel tumor-promoting function of HSATII RNA, highlighting its propensity to form dsRNA through interactions with STRBP. Although further investigations are required to unravel the detailed mechanisms, our findings enhance our understanding of the biological significance of aberrant expression of satellite repeats in pancreatic cancer. Subsequent studies involving mouse models or clinical samples would provide valuable insights into the clinical implications of our observations.

## Experimental procedures

### Cell culture

Human pancreatic cancer cell lines MiaPaCa-2, Panc-1, and BxPC-3 were obtained from the American Type Culture Collection (ATCC), and cultured in Dulbecco's modified Eagle's medium (DMEM) supplemented with 10% fetal bovine serum (FBS), DMEM with 20% FBS, or RPMI medium with 10% FBS, respectively. To develop tumor spheroids for 3D culture, Panc-1 and BxPC-3 cells were seeded on ultra-low attachment dishes (Corning Inc) and cultured with 3D Tumorsphere Medium XF (Promo Cell). The human embryonic kidney cell line HEK293T was purchased from System Biosciences (SBI) and cultured in DMEM with 10% FBS. All cells were incubated at 37 °C in an atmosphere of 20% O_2_ and 5% CO_2_.

### Subcutaneous xenograft model

For allogeneic transplantation, 1 × 10^5^ MiaPaCa-2 cells or 1 × 10^6^ Panc-1 cells in 100 μl of DMEM were mixed with 100 μl of Matrigel basement membrane matrix, LDEV-Free (Corning), and immediately injected subcutaneously into the backs of female *BALB/cAJcl-Foxn1*^*nu*^ mice (CREA Japan Inc). The resulting tumors were excised after 4 weeks.

### RNA extraction

RNA was extracted from cells using RNeasy Mini Kit (QIAGEN) with RNase-Free DNase Set (QIAGEN) to remove DNA according to the manufacturer’s protocol. RNA was extracted from xenografts minced in ISOGEN (Nippon Gene) followed by DNase digestion with the RNase-Free DNase Set.

### Northern blotting

Northern blotting was performed as previously described (29). Briefly, total RNA was separated on a Mops/formaldehyde-agarose gel at 100 V for 50 min and transferred hydrostatically to Hybond N+ membranes (GE HealthCare). The membranes were UV-crosslinked and prehybridized in ULTRAhyb-Oligo Hybridization Buffer (Ambion, Thermo Fisher Scientific). Hybridization was performed for 16 h at 42 °C in a hybridization buffer containing digoxigenin-labeled RNA probes denatured for 5 min at 95 °C. The membranes were stringently washed, and the bound probe was visualized using CDP-star (Roche) according to the manufacturer’s protocol. The digoxigenin-labeled RNA probes anti-HSATII S: 5′-CATTCGATTCCATTCGATGAT-3′ and anti-HSATII AS: 5′-ATCATCGAATGGAATCGAATG-3′ were synthesized artificially (Integrated DNA Technologies).

### Immunohistochemistry

Immunohistochemistry was performed as previously described (45). Briefly, fixed paraffin-embedded tissue microarrays PA243, PA804b, and PA811 (US Biomax) were deparaffinized, and incubated in Target Retrieval Solution (Dako Corp) buffer for 30 min at 98 °C for antigen retrieval. Endogenous peroxidase activity was blocked by incubation in 3% hydrogen peroxide buffer for 10 min. To minimize nonspecific background staining, slides were blocked in 10% normal goat serum (Dako) for 30 min at 20 °C and incubated for 16 h at 4 °C with anti-J2-dsRNA antibody (Scicons) diluted in 2% bovine serum albumin/phosphate-buffered saline with polysorbate 20. Slides were incubated with biotinylated rabbit antibody (BD Pharmingen) for 20 min at 20 °C. After 30 min of adding streptavidin–horseradish peroxidase (BD Pharmingen), the tissues were developed with 3,3′-diaminobenzidine (DAB) substrate (Vector Laboratories Inc) according to the manufacturer’s protocol and counterstained with hematoxylin.

### Plasmid construction

For bidirectional HSATII RNA overexpression, a 170 bp segment of the consensus HSATII sequence (HSATII Fw) registered in the Dfam database (https://www.dfam.org/) and a complementary sequence (HSATII Rv) were synthesized and cloned into pEX-A2J2 vector (Eurofins Scientific). The pBI-dsHSATII plasmid was constructed using the pBI-CMV1 vector (Clontech Laboratories Inc) containing divergent CMV promoters. The HSATII Fw and HSATII Rv sequences were subcloned into the pBI-CMV1 vector at HindIII and BglII, respectively. For bidirectional vectors expressing other repetitive arrays, HERVH, AluSX, and L1M1, a 200 bp segment from each consensus sequence registered in the Dfam database was synthesized and cloned into pEX-A2J2 vector (Eurofins Scientific). The sequence of each segment is shown in Table S2. The repeat sequence was cut by BamHI and EcoRV and subcloned into the mcs1 site of the pBI-CMV1 vector. Subsequently, the same repeat sequence digested by XbaI and EcoRI was subcloned into the mcs2 site.

The shRNA oligonucleotides were annealed and cloned into a pLKO.1-Puromycin lentiviral vector (Addgene). The sequences used for the respective target genes were as follows: shScramble: 5′-CCTAAGGTTAAGTCGCCCTCG-3′, shSTRBP: 5′-CGCTTTGTAATGGAGGTAGAA-3′, shCLSTN1 long1: 5′- CAGGAGTTGAAAATGACAATG-3′, shCLSTN1 long2: 5′-CAATGAAACTGAGCCTGTGAC-3′, shCLSTN1 long3: 5′-GAAACTGAGCCTGTGACTGTG-3′, shCLSTN1 short1: 5′-GCTTGCTGGCAAGGTGGCGAC-3′, shCLSTN1 short2: 5′- GCTGGCAAGGTGGCGACCTGC-3′, and shCLSTN1 short3: 5′-GGCAAGGTGGCGACCTGCACA-3’.

The gRNA oligonucleotides were annealed and subcloned into the Lenti-CRISPR-v2-Puromycin vector (Addgene). The gRNA sequence was STRBP KO: 5′-CATGCCAAATGGTTTCAGGT-3′.

For STRBP overexpression, the Halo Tag-STRBP-complementary DNA (cDNA) vector (FHC01534) was purchased from Kazusa Genome Technology. Using this plasmid as a template, the PCR-amplified STRBP open reading frame was subcloned into the pLVSIN-EF1α-MCS-Puro vector (Takara Bio). The lentiviral vectors expressing HA-tagged STRBP and its deletion mutant were constructed by VectorBuilder.

### Transfection and lentivirus transduction

The plasmids were transiently transfected using Effectene Transfection Reagent (QIAGEN). Briefly, 24 h after seeding 1 × 10^6^ cells in a 6 cm dish, 1 μg of template plasmid and Effectene Transfection Reagents were added to the dish, according to the manufacturer’s protocol. Monoclonal cells were isolated from single-cell clones of pBI-Empty, pBI-HSATII-Fw, and pBI-dsHSATII vectors. Lentivirus Packaging System (SBI) was used to generate stably expressing polyclonal cells according to the manufacturer’s protocol. Briefly, the plasmid and pPACKH1 packaging plasmid mix were transfected into HEK293T cells using Effectene Transfection Reagent. After 48 h, the viruses were concentrated in the culture media using PEG-it Reagent (SBI). The centrifuged pellet was resuspended in 1 × PBS, and aliquots were stored at −80 °C. Cells were infected with virus using polybrene reagent (Santa Cruz Biotechnology), followed by selection with puromycin.

### RNase A treatment

To confirm dsRNA formation by RNase A digestion, 50 μg of total RNA extracted from Panc-1 cells expressing bidirectional HSATII was incubated with 0.25 μl of RNase A/T1 Mix (Thermo Fisher Scientific) in digestion buffer (3.5 M NaCl) for 5 min at 20 °C. dsRNA was purified with phenol–chloroform isoamyl alcohol (25:24:1) and chloroform.

### Reverse transcription and quantitative PCR (RT-qPCR)

Reverse transcription was performed using the SuperScript III First-Strand Synthesis Supermix (Thermo Fisher Scientific), and quantitative PCR was performed using the Thunderbird SYBR qPCR Mix (Toyobo Co) and StepOnePlus real-time PCR system (Life Technologies). The relative expression levels were normalized to the expression levels of *ACTB* mRNA and calculated using the delta-delta Ct method. The primers used in this study are listed in Table S3.

### Immunofluorescence cytochemistry

Bidirectional vectors, pBI-empty, pBI-HSATII Fw, and pBI-dsHSATII, were transiently transfected into Panc-1 cells using Effectene Transfection Reagents in 4-well chamber glass slides (IWAKI). Following 24 h incubation, cells were fixed with 4% paraformaldehyde for 10 min at 4 °C and subsequently permeabilized with 0.01% Triton-X for 20 min at 20 °C. Samples underwent incubation with anti-J2-dsRNA antibody diluted with 150 μl of Can Get Signal immunostain Solution B (Toyobo Co), overnight, at 4 °C. The secondary antibody, Goat anti-Mouse immunoglobulin G (IgG) Alexa Fluor 555 (A-21422, Invitrogen, Thermo Fisher Scientific), diluted with 150 μl of Can Get Signal immunostain Solution B was applied for 30 min at 20 °C. The slides were washed four times with 1 × PBS, and mounted with VECTASHIELD Mounting Medium (Vector Laboratories).

### Western blotting and antibodies

Western blotting was performed as previously described (29). Briefly, total lysate samples were separated in 10 to 20% gradient polyacrylamide gels (Fujifilm Wako Pure Chemicals) and transferred to immobilon-P membranes, (polyvinylidene fluoride; Merck). After blocking with 5% dry milk, the membranes were probed with the appropriate primary antibodies diluted in Immunoshot Reagent 1 (Cosmo Bio) for 16 h at 4 °C. The corresponding horseradish peroxidase (HRP)–conjugated secondary antibodies (GE HealthCare) were then added. Bound antibodies were detected using the Immunostar LD Reagent (Wako Pure Chemical Industries). The antibodies used in this study are listed in Table S4.

### Invasion assay

Invasion assay was performed using Corning BioCoat Matrigel Invasion Chambers with an 8.0 μm PET Membrane in 24-well plates (Corning) with 5 × 10^5^ cells seeded in the upper chamber of the assay plate. FBS was added to the feeder tray and the assay as a chemoattractant, and the assay plate was cultured for 48 h. Invasive cells were stained using a Diff-Quick III Stain Kit (Sysmex Corp) and counted under a microscope. Data are shown as the mean number of invading cells from five randomly selected fields per sample.

### Flow cytometry

Panc-1 cells (8 × 10^5^) seeded in 6 cm plates were collected using a cell scraper with l × PBS containing 0.5% bovine serum albumin. The cells were washed twice with l × PBS and resuspended in 100 μl of Cell Staining Buffer (BioLegend). Subsequently, 2 μl of fluorescein isothiocyanate-conjugated anti-human CD133 antibody (130-113-673, Miltenyi Biotec) or Alexa Fluor 488–conjugated anti-CD44 antibody (103016, BioLegend) was added and incubated for 20 min at 4 °C in the dark. Cells were washed twice and resuspended in 750 μl of Cell Staining Buffer. Samples were analyzed using the EasyCyte Mini Flow Cytometer (Luminex) and FlowJo software (BD Bioscience, version 10, https://www.flowjo.com/).

### RNA pull-down assay

To perform RNA pull-down assay, dsRNA oligonucleotides were artificially synthesized by *in vitro* transcription using a MEGAscript T7 Transcription Kit (Ambion, Thermo Fisher Scientific). A 170-bp of the consensus HSATII sequence was subcloned into the pcDNA 3.1 (−) and (+) plasmids (Invitrogen), and used as a template for *in vitro* transcription regulated by the T7 promoter. After denaturing followed by gradient cooling, the transcribed products were annealed between complementary strand sequences. The annealed oligonucleotides were treated with S1 nuclease (Takara Bio) for 15 min at 20 °C, according to the manufacturer’s protocol, to digest the flanking unannealed parts to form a complete duplex of 170 bp. dsRNA was purified with phenol-chloroform-isoamyl alcohol (25:24:1) and chloroform.

To identify protein that binds to dsHSATII RNA *in vitro*, an RNA pull-down assay was performed using Pierce Magnetic RNA-Protein Pull-Down Kit (Thermo Fisher Scientific) according to the manufacturer’s protocol. Briefly, biotinylated dsHSATII and HSATII Fw RNA oligonucleotides were synthesized by *in vitro* transcription and bound to streptavidin magnetic beads. They were mixed with Panc-1 cell lysates and incubated for 16 h at 4 °C. Binding proteins were precipitated and separated on a 10 to 20% gradient polyacrylamide gel by SDS-PAGE with 5% input, followed by silver staining. Three bands, specifically detected in the dsHSATII lanes, were excised from the gel and subjected to LC-MS/MS analysis.

### Mass spectrometry

The gel pieces were washed twice with 100 mM bicarbonate in acetonitrile, and the proteins were digested with trypsin for 20 h at 37 °C. After adding 0.1% formic acid to the supernatant, the peptides were analyzed by LC-MS/MS with an Easy-nLC 1200 System (Thermo Fisher Scientific) and a Q Exactive plus (Thermo Fisher Scientific). The resulting tandem mass spectrometry dataset was analyzed using the Mascot software program (Matrix Science; version 2.7.0, https://www.matrixscience.com/). Mascot was set up to search the SwissProt_2020_06 database (selected for *Homo sapiens*, unknown version, 20395 entries) assuming the digestion enzyme strict trypsin. Mascot was searched with a fragment ion mass tolerance of 0.020 Da and a parent ion tolerance of 5.0 PPM. Scaffold (version Scaffold_5.2.2, Proteome Software Inc, https://www.proteomesoftware.com/products/scaffold-5) was used to validate MS/MS based peptide and protein identifications.

### RNA immunoprecipitation

To evaluate the expression profiles of dsRNA in Panc-1 xenografts, an RNA immunoprecipitation assay was performed using anti-J2-dsRNA antibody, according the methodology in a previous paper (46). Specifically, 25 μl of Pierce Protein A/G magnetic beads (Invitrogen) was coupled with 10 μl of anti-J2-dsRNA antibody or Normal mouse IgG in 100 μl of NET-2 buffer (50 mM Tris–HCl pH 7.4, 150 mM NaCl, 1 mM MgCl2, and 0.5% [v/v] NP-40) for 1 h at 20 °C. Subsequently, 30 mg of xenograft tissue, washed with ice-cold PBS twice, was homogenized mechanically in 1 ml of NP-40 lysis buffer (50 mM Tris–HCl pH 7.4, 150 mM NaCl, 1 mM EDTA, 0.5% NP-40, 125 nM RNase OUT [Invitrogen], and Complete Protease Inhibitor cocktail [Sigma-Aldrich]) for 10 min at 4 °C, followed by sonication with Sonifier 250 (Branson Ultrasonics). After centrifugation at 15,000*g* for 10 min at 4 °C, the supernatant was collected as the lysate. Beads bound with anti-J2-dsRNA antibody were washed twice and pelleted in a 2 ml tube. Subsequently, 350 μl of the lysates diluted with 1400 μl NET-2 buffer was applied to the tube and incubated with gentle rotation overnight at 4 °C. The bound RNA was washed twice with High Salt Buffer (50 mM Tris–HCl pH 7.4, 1 M NaCl, 1 mM EDTA, 1% NP-4 0.01% SDS, and 0.5% sodium deoxycholate), followed by two additional washes with NET-2 buffer. RNA was extracted from the bead pellet using 1 ml of the ISOGEN solution, followed by genomic DNA digestion with ezDNase Enzyme (Invitrogen).

To examine the intracellular binding of STRBP protein and dsHSATII RNA, RNA immunoprecipitation was performed using the RNA immunoprecipitation (RIP)-Assay Kit (MBL) according to the manufacturer’s protocol. Briefly, 2 × 10^6^ HEK293T cells were seeded on a 10 cm dish, and the pBI-dsHSATII vector was transfected into the cells using the Effectene Transfection Reagent. After 48 h, the cells were scraped and lysed with M-PER Mammalian Protein Extraction Reagent (Thermo Fisher Scientific), followed by the addition of precleared protein A/G beads conjugated to anti-STRBP antibody (Proteintech) or control rabbit IgG. The bound RNA was washed and isolated using a precipitation procedure. Immunoprecipitated RNA and 5% input were reverse transcribed to cDNA using ReverTra Ace (Toyobo Co) with DNase I treatment. The relative amount of immunoprecipitated RNA was quantitated to the qPCR with normalization by input sample, and precipitated STRBP was confirmed by Western blotting.

For the assessment of the binding efficacy of STRBP deletion mutant, 2 × 10^6^ HEK293T cells were seeded on a 10 cm dish, and either the HA-STRBP or HA-STRBP ΔdsRBM vector were cotransfected with the pBI-dsHSATII vector into the cells using the Effectene Transfection Reagent. After 48 h, the cells were scraped and lysed with M-PER Mammalian Protein Extraction Reagent. Samples were incubated with anti-HA-tag mAb-Magnetic Beads (M180-11, MBL) or control mouse IgG bound to Protein A/G magnetic beads overnight at 4 °C. After four times washing, bound RNA was isolated and subjected to RT-qPCR. Precipitated HA-tagged STRBP was confirmed by Western blotting using anti-HA antibody.

For the immunoprecipitation of Panc-1 cells, the lentiviral expression vector for expressing HA-tagged STRBP was transduced into Panc-1 dsHSATII cells, followed by puromycin selection. The cells (5 × 10^6^) seeded in 10 cm dish were scraped and lysed with M-PER Mammalian Protein Extraction Reagent, followed by the same immunoprecipitation procedures used for HEK293T cells.

### Semiquantitative PCR and quantitative PCR of splicing isoforms

Total RNA was reverse transcribed using SuperScript III reverse transcriptase (Invitrogen), and cDNA was amplified using Q5 High-Fidelity DNA polymerase (New England Biology). PCR products were separated by electrophoresis on a 2% agarose gel and visualized using an ethidium bromide solution. Each PCR band was quantitated using ImageJ software (https://imagej.net/ij/).

For RT-qPCR analyses, extracted RNA was reverse transcribed using the SuperScript III First-Strand Synthesis Supermix, and subsequently subjected to qPCR with specific primers designed for each isoform of CLSTN1, ARAP1, and ATP5C1. The primers used in this study are listed in Table S3.

### Statistical analysis

Statistically significant differences were determined using Student’s *t* test or Fisher’s exact test between two groups, and one-way Analysis of Variance (ANOVA) followed by Tukey’s multiple comparisons test or Bonferroni test for multigroup datasets. Statistical significance was set at *p* < 0.05. All data analyses were performed using GraphPad Prism 9.5 (GraphPad Software, https://www.graphpad.com/).

### Study approval

Animal experiments were approved by the Internal Ethics Committee for Animal Experimentation (approval number #H21-113, #P21-047) and conducted per the guidelines for the Care and Use of Laboratory Animals of Graduate School of Medicine, the University of Tokyo (Tokyo, Japan).

## Data availability

All representative data are contained within the article.

## Supporting information

This article contains supporting information.

## Conflict of interest

The authors declare no competing interests with the contents of this article.
