# Supplementary material for: Satellite double-stranded RNA induces mesenchymal transition in pancreatic cancer by regulating alternative splicing
Source: J Biol Chem. 2024 Feb 10;300(3):105742. doi: 10.1016/j.jbc.2024.105742 (PMC10943486; doi:10.1016/j.jbc.2024.105742)
Supplement: Supporting Figures S1–S5 and Tables S1–S4 legends [file mmc1.pdf]

Supplementary Information

Supplementary Figures and legends (Figures S1-S5)

Fig. S1. Overexpression of dsHSATII RNA induced mesenchymal-like phenotypes in pancreatic cancer cell lines.

Fig. S2. Establishment of Panc-1 cells stably overexpressing dsRNAs derived from repetitive elements.

Fig. S3. STRBP is an RNA binding protein bound to dsHSATII RNA.

Fig. S4. The population of CD133-positive cells and the expression levels of CD44 were elevated by STRBP depletion and downregulated by STRBP overexpression.

Fig. S5. The splicing patterns shifted in Panc-1 cells cultured in 3D settings or xenografts without altering the expression of STRBP.

Supplementary Tables (Table S1-S4)

Table S1. List of candidate proteins binding to dsHSATII RNA identified by LC-MS/MS analyses.

Table S2. List of inserted sequences of repetitive elements in bidirectional expression vectors.

Table S3. List of primers used for RT-qPCR analyses.

Table S4. List of antibodies used for western blotting.

Supplementary Figure 1

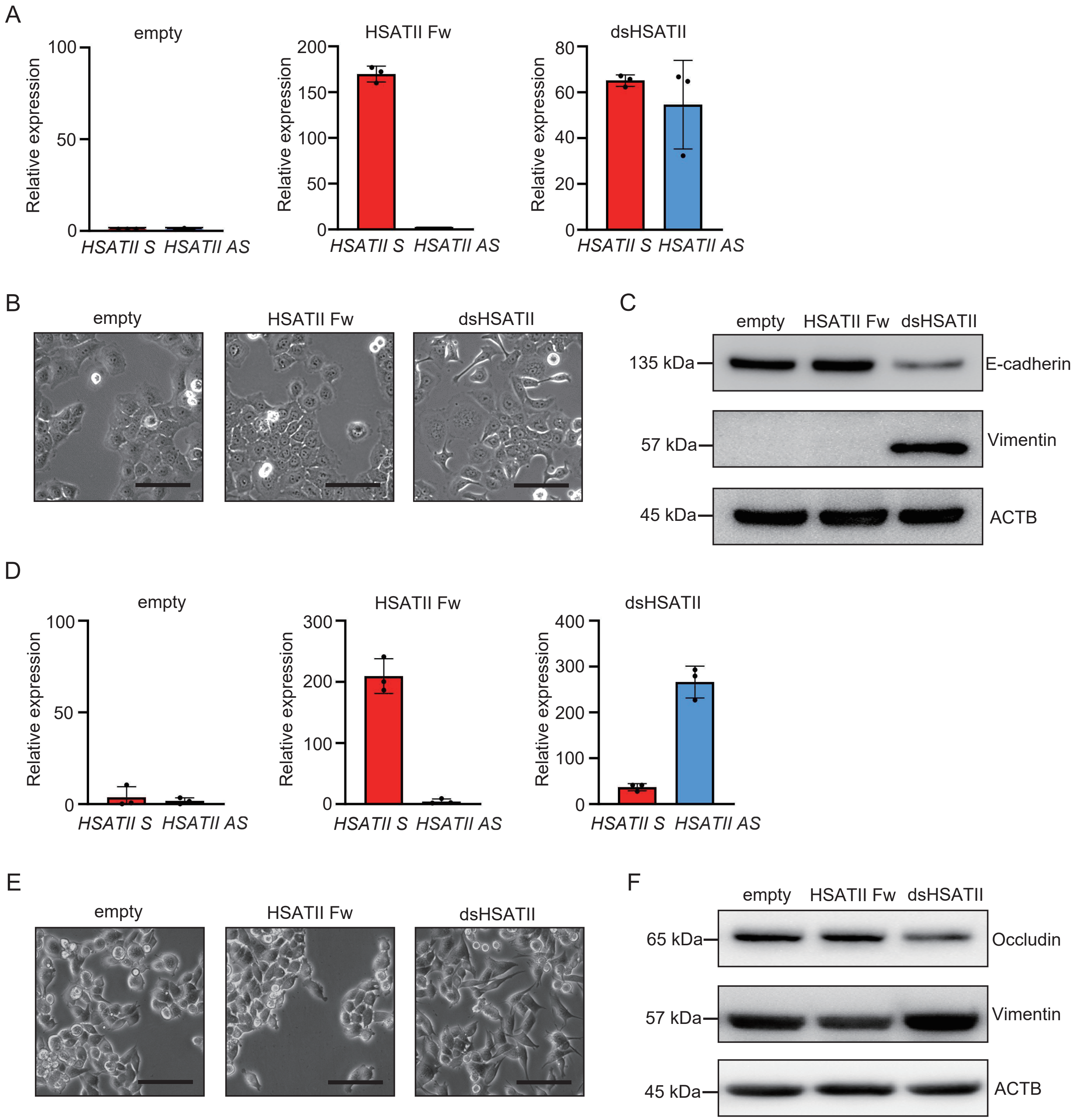

Supplementary Figure 2

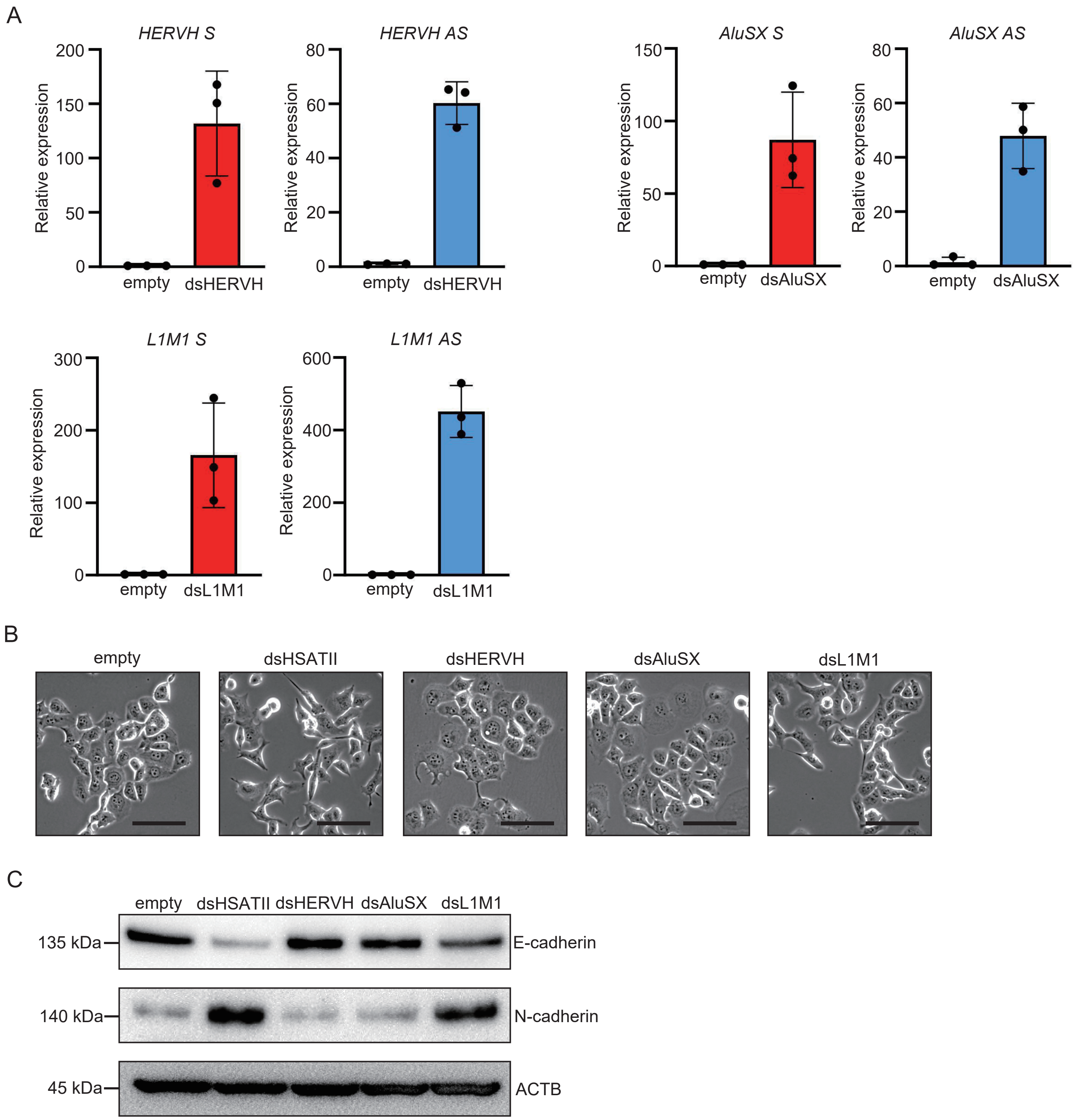

Supplementary Figure 3

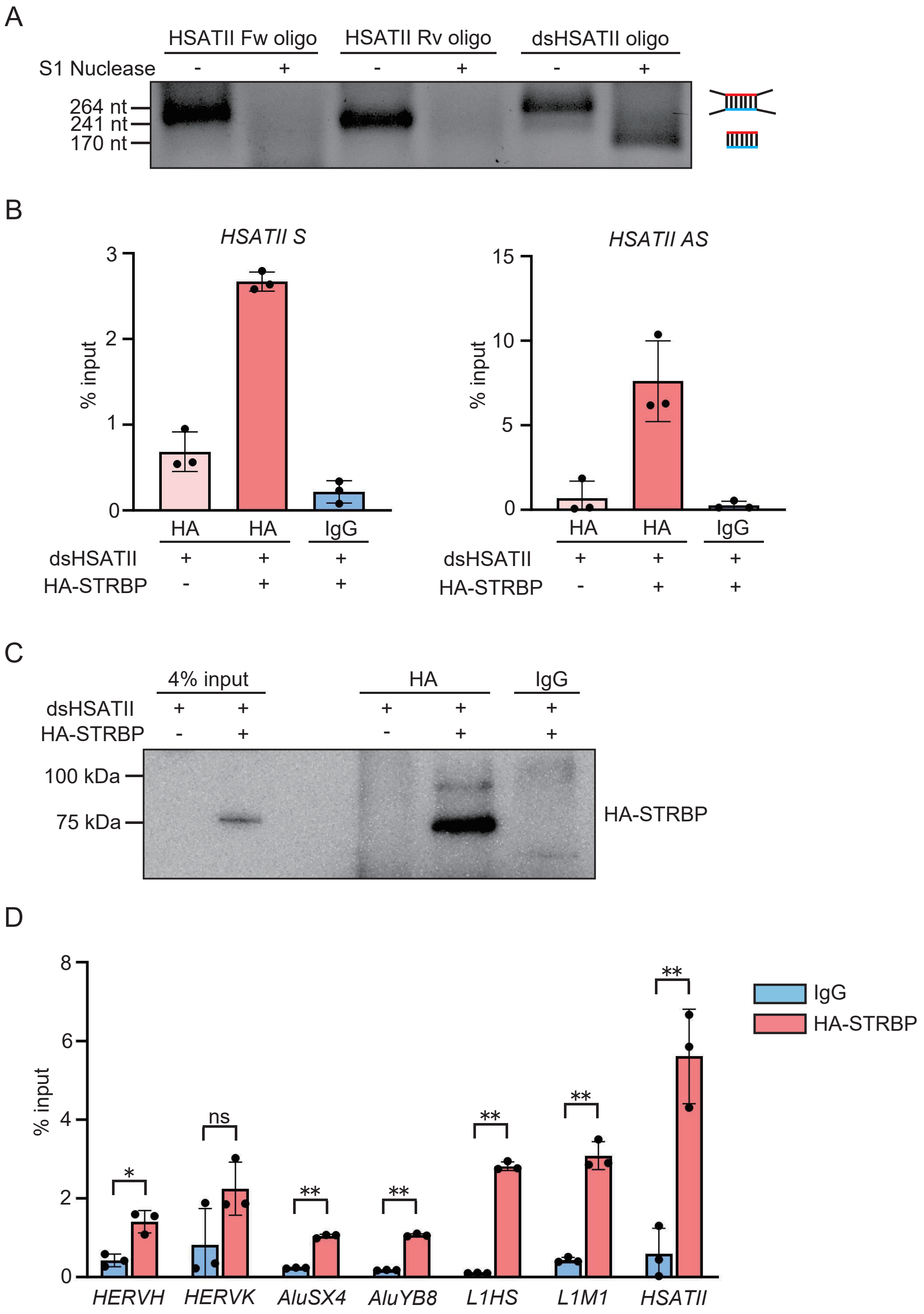

Supplementary Figure 4

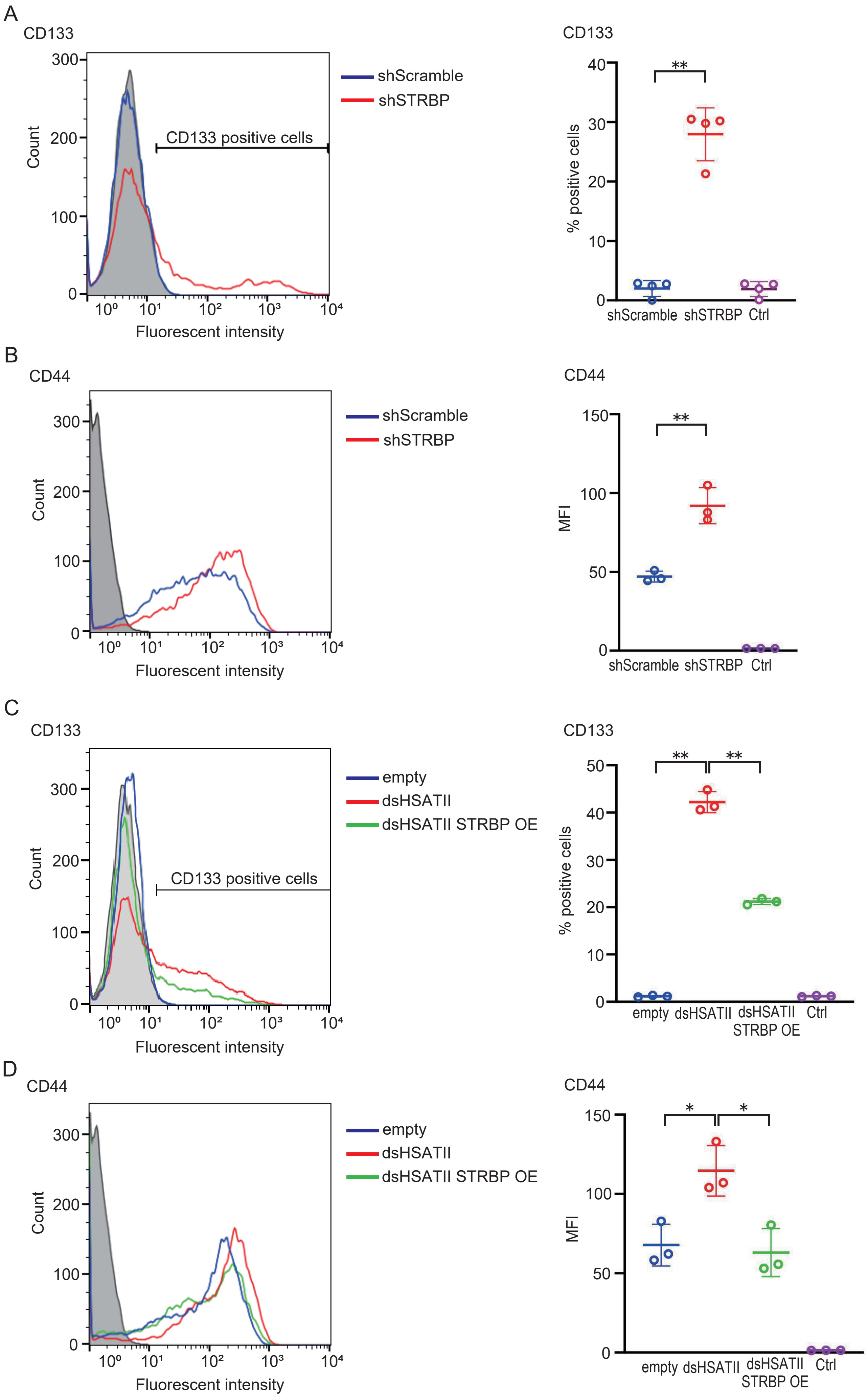

Supplementary Figure 5

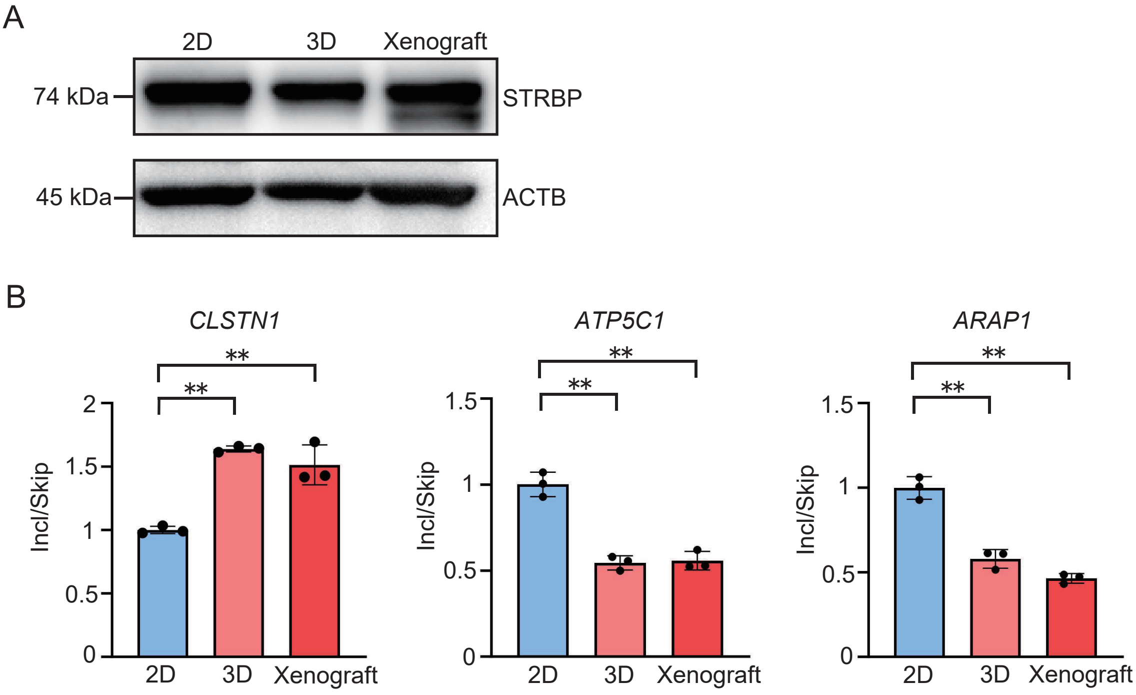

Supplementary Figure Legends

Figure S1. Overexpression of dsHSATII RNA induced mesenchymal-like phenotypes in pancreatic cancer cell lines.

(A) Relative expression levels of both strands of HSATII RNA in BxPC-3 cells stably expressing HSATII Fw and dsHSATII RNA. Data are shown as mean ± S.D. of triplicate experiments. **(B)** Representative morphological images of BxPC-3 cells stably expressing HSATII Fw and dsHSATII RNA. Scale bars: 50 μm. **(C)** Western blotting images of EMT markers in BxPC-3 cells. **(D)** Relative expression levels of both strands of HSATII RNA in MiaPaCa-2 cells stably expressing HSATII Fw and dsHSATII RNA. Data are shown as mean ± S.D. of triplicate experiments. **(E)** Representative morphological images of MiaPaCa-2 cells stably expressing HSATII Fw and dsHSATII RNA. Scale bars: 50 μm. **(F)** Western blotting images of EMT markers in MiaPaCa-2 cells. ACTB was used as a loading control.

Figure S2. Establishment of Panc-1 cells stably overexpressing dsRNAs derived from repetitive elements.

(A) Relative expression levels of each strand of repetitive sequences in Panc-1 cells stably expressing dsRNAs of HERVH, AluSX, or L1M1. RT-qPCR analyses were performed using specific primer pairs for both strands. Data are shown as mean ± S.D. of triplicate assay. **(B)** Representative morphological images of Panc-1 cells expressing dsRNAs. Scale bars: 50 μm. **(C)** Western blotting images of EMT markers in Panc-1 cells expressing dsRNAs. ACTB was used as a loading control.

Figure S3. STRBP is an RNA binding protein bound to dsHSATII RNA.

(A) RNA oligonucleotides synthesized by in vitro transcription were treated with S1 nuclease. Mops-formaldehyde agarose gel electrophoresis showed that dsHSATII oligonucleotides were protected from S1 nuclease, while flanking non-complementary parts were digested. **(B)** RNA immunoprecipitation assay was conducted using anti-HA-tag antibody on Panc-1 cells stably expressing dsHSATII RNA and HA-STRBP. Both strands of HSATII RNA were assessed by RT-qPCR and plotted as a percentage of the input. **(C)** Western blotting image of the immunoprecipitated HA-STRBP. **(D)** The enrichment of repetitive sequences in the immunoprecipitates in Panc-1 cells using anti-HA-tag antibody. Data are shown as mean ± S.D. of triplicate assay of RT-qPCR. *: $p < 0.05$, **: $p < 0.01$, ns: not significant (Student's t-test).

Figure S4. The population of CD133-positive cells and the expression levels of CD44 were elevated by *STRBP* depletion and downregulated by STRBP overexpression.

(A) (C) The expression of CD133 was evaluated by flow cytometry in Panc-1 STRBP KD cells **(A)** and Panc-1 dsHSATII-STRBP OE cells **(C)**. Representative histogram was shown at the left panel and the percentage of CD133-positive cells was plotted in the right panel. Data are shown as mean ± S.D. from quadruplicate assays. **(B) (D)** The expression levels of CD44 were evaluated by flow cytometry in Panc-1 STRBP KD cells **(B)** and Panc-1 dsHSATII-STRBP OE cells **(D)**. Mean fluorescence intensities (MFI) were plotted in the right panel. Data are shown as mean ± S.D. from quadruplicate assays. *: $p < 0.05$, **: $p < 0.01$ (Student's *t*-test and one-way ANOVA followed by Tukey's multiple comparisons test).

Figure S5. The splicing patterns shifted in Panc-1 cells cultured in 3D settings or xenografts without altering the expression of STRBP.

(A) Western blotting images for STRBP in Panc-1 cells cultured under 2D, 3D, or xenograft condition. ACTB was used as the loading control. **(B)** Changes of alternative splicing pattern in Panc-1 under each culture conditions. RT-qPCR was performed using isoform specific primers and the ratio of exon inclusion to exon skipping was plotted as mean ± S.D. of triplicate assay. **: $p < 0.01$ (one-way ANOVA followed by Tukey's multiple comparisons test).
